# Immunoreactive Proteins in the Esophageal Gland Cells of Anisakis Simplex Sensu Stricto Detected by MALDI-TOF/TOF Analysis

**DOI:** 10.3390/genes11060683

**Published:** 2020-06-22

**Authors:** Lee Robertson, Susana C. Arcos, Sergio Ciordia, Noelia Carballeda-Sanguiao, María del Carmen Mena, Isabel Sánchez-Alonso, Miguel Gonzalez-Muñoz, Mercedes Careche, Alfonso Navas

**Affiliations:** 1 Department of Biodiversity and Evolutionary Biology, Museo Nacional de Ciencias Naturales, CSIC, calle José Gutiérrez Abascal 2, 28006 Madrid, Spain; scobacho@mncn.csic.es; 2 Departamento de Protección Vegetal, INIA, Ctra La Coruña Km 7′5, 28040 Madrid, Spain; 3 Unidad de Proteómica Centro Nacional de Biotecnología, CSIC, calle Darwin 3, Campus de Cantoblanco, 28049 Madrid, Spain; sciordia@cnb.csic.es (S.C.); mcm.mena1@gmail.com (M.d.C.M.); 4 Instituto de Ciencia y Tecnología de Alimentos y Nutrición, CSIC, Calle José Antonio Novais, 10, 28040 Madrid, Spain; noeliacarba@hotmail.com (N.C.-S.); isabel.sanchez@csic.es (I.S.-A.); mcareche@ictan.csic.es (M.C.); 5 Servicio de Immunología, Hospital Universitario La Paz. Paseo de la Castellana, 261, 28046 Madrid, Spain; mgonzalez_munoz@hotmail.com

**Keywords:** *Anisakis simplex*, allergens, esophageal glands, proteomics, excretion/secretion

## Abstract

In plant and animal nematode parasites, proteins derived from esophageal gland cells have been shown to be important in the host-nematodes relationship but little is known about the allergenic potential of these proteins in the genus *Anisakis*. Taking into account the increase of anisakiasis and allergies related to these nematodes, immunoreactive properties of gland cell proteins were investigated. Two hundred ventricles were manually dissected from L3 stage larvae of *Aniskakis simplex s.s*. to allow direct protein analysis. Denaturing gel electrophoresis followed by monochromatic silver staining which revealed the presence of differential (enriched) proteins when compared to total nematode extracts. Such comparison was performed by means of 1D and 2D electrophoresis. Pooled antisera from *Anisakis* spp.-allergic patients were used in western blots revealing the presence of 13 immunoreactive bands in the ventricular extracts in 1D, with 82 spots revealed in 2D. The corresponding protein bands and spots were excised from the silver-stained gel and protein assignation was made by MALDI-TOF/TOF. A total of 13 (including proteoforms) were unambiguously identified. The majority of these proteins are known to be secreted by nematodes into the external environment, of which three are described as being major allergens in other organisms with different phylogenetic origin and one is an *Anisakis simplex* allergen.

## 1. Introduction

Humans are incidental hosts of *Anisakis simplex* and become infected through eating live L3 larvae found in raw or undercooked fish and cephalopod meat, developing the disease known as anisakiosis or anisakiasis. The main symptoms are epigastralgy, vomiting, nausea, abdominal pain, and diarrhea of differing intensity that generally appear 24 h after intake of infected sea products [1]. Additional symptoms associated with parasite exposure are IgE-mediated hypersensitivity, angioedema, urticaria, and anaphylaxis [2,3]. 

However, the allergic reaction to *Anisakis* is not always directly related to larvae ingestion. It has been reported that some people sensitized to *A. simplex s.l*. can show symptoms after consuming fish that has been properly frozen, cooked, and even processed suggesting the presence of allergens excreted or secreted by the nematodes in the fish host [4,5,6]. On the other hand, immunoblot studies using sera from *Anisakis*-sensitized patients have proved that some allergenic proteins of *Anisakis* are not inactivated after thermal treatments [5,6,7]. To date there are 19 described allergens in *Anisakis* [8]; however, a recent proteomic study combining 2D gel analysis and western blotting described 28 immunoreactive proteins present in of the species complex (*A. simplex*, *A. pegreffii*, and their hybrid), including intraspecies variations which could be assessed as potential allergens. None of these new potential allergens are considered as members of the protein secretome and were recovered in the whole-body extract of assayed species complex [9].

Parasitic nematodes secrete/excrete proteins which are thought to be involved in the host-parasite relationship and are often found as circulatory antigens [10]. Secretions and excretions can arise from a number of nematode structures which are open to the external environment including the excretory pore (E/S cells), cuticle, amphids, esophageal gland cells, and anus. The importance of secreted proteins in *Anisakis simplex* as more potent allergens than somatic ones was highlighted [11] by means of purifying secreted proteins in a nematode culture medium; however, no distinction was made between secreted and excreted proteins. In this study, we analyze and characterize the immuno reactive proteins (potential allergens) from *A. simplex s.s*. obtained by direct analysis of the esophageal ventricle glands.

## 2. Materials and Methods

### 2.1. Parasite Material

Third stage juveniles (L3 larvae) were collected from the kidney of *Merluccius merluccius* (European hake) and the species was molecularly determined as *A. simplex*. *s.s*. following the diagnostic keys based on PCR-RFLP analysis of the nuclear ribosomal marker internal transcribed spacer (ITS) [12]. Two different extraction buffers using total animal body for control were used to prepare total nematode protein extracts for selecting the proper method in order to proceed with the proteomic characterization of immunoreactive esophageal ventricle gland proteins: a) extraction 1 (E1) was denaturing (7 M urea, 2 M thiourea, Triton X-100 2%, 40 mM DTT) and b) extraction 2 (E2) was a not denaturing method (0.1 M sodium phosphate buffer). Ten nematodes were crushed with a mortar and pestle in either the denaturing buffer (E1) or the 0.1 M sodium phosphate buffer (E2). For esophageal ventricle glands, two hundred gland cells (GC) of the L3 larvae were dissected under a binocular microscope. 

Then, gland cells were collected in a total volume of 100 μL of the sodium phosphate buffer (0.1 M Na_2_H_3_PO_4_, pH 7.2), and the proteins extracted by gently crushing the gland cells with a micropestle. The extracted proteins were then divided into two replicates (GC1 and GC2). In all cases, the nematode material was centrifuged at 13,000 rpm for 2 min to pellet insoluble material. The supernatant was removed to a separate tube and quantified either using the RC/DC Protein Assay kit (Biorad, Hercules, CA, USA) and the method of Lowry et al. [13] and stored at −20 °C until required, as was the case of proteins extracted in phosphate buffer. 

### 2.2. 1D SDS-PAGE 

Before electrophoresis running, samples were prepared for SDS-PAGE by heating the proteins in an equal volume of 2× sample buffer (62 mM Tris HCl pH 6.8, 10% glycerol, 2% SDS, 5% B-mercaptoethanol, and 0.25% Bromofenol blue) for 2 min. Five µg of protein were loaded into each well and separated on a 10%–20% gradient precast gels in Tris/Glycine running buffer (25 mM Tris, 192 mM glycine, 0.1% SDS, pH 8.3) using the Criteron system (Bio-Rad). Gels were run for approx. 1 h at a constant voltage of 150 V. 

### 2.3. Two-Dimensional Gel Electrophoresis 

Products for 2-D electrophoresis were supplied by GE Healthcare Life Sciences (www.ge.com). The extracted proteins by phosphate buffer were cleaned up in order to improve resolution of 2-D electrophoresis gels; the chemicals used to clean up proteins and their quantification were from Bio-Rad (www.Bio-Rad.com). The other need chemicals were analytical grade from Sigma-Aldrich (http://www.sigmaaldrich.com). 200 μL of rehydration buffer (8 M urea, 1.5 M Thiourea, 2% (v/v) Triton-100, 0.5% (v/v) IPG buffer 3–10, and 0.01% (w/v) bromophenol blue) were used to rehydrate 11 cm IPG strips with a non-linear gradient (pH 3–11 NL, GE Healthcare) before to perform isoelectric focusing (IEF). Approximately 60 μg of total protein was used for each gel. IEF was performed on the Ettan™ IPG phor II™ system (GE Healthcare) with current restricted to 50 μA per strip. Running condition was 20 °C with a voltage of 150 V for 2 h, 500 V for 1 h, 1000 V for 1 h, 8000 V for 2.5 h, 8000 V for 0.5 h, and a 500 V hold when required. Before the second dimension, the IPG strips were gently soaked in equilibration solution (6 M urea, 50 mM Tris-HCI buffer pH 8.8, 29.3% v/v glycerol, 2% w/v SDS, and 0.002% bromophenol blue) containing 1% w/v DTT for 15 min followed by a further incubation in equilibration solution containing 2.5% (w/v) iodoacetamide for 15 min. Second-dimension gel electrophoresis was carried out on 10%–20% polyacrylamide Ready Gel precast gels using the CRITERION™ Cell (Bio-Rad) for 11 cm strips. Equilibrated strips were placed onto gels to perform the SDS-PAGE at a constant temperature of 20 °C. The separation was carried out at 12.5 mA per gel for 15 min and then 25 mA per gel until the bromophenol blue marker reached the bottom of the gel. Electrophoresis was performed simultaneously on two gels which were matched for each sample. Gels were developed following the silver stain procedure. After rinsing, gels were immersed in preserving solution (30% v/v ethanol, 4.6% v/v glycerol) for 1 h before proceeding to image acquisition and analysis.

### 2.4. Silver Staining

Gels were silver stained as described by Wray et al. (1981) [14]. Briefly, gels were fixed in 50% methanol and then immerse in 100 mL of stain solution (containing 5 mL of 4% NaOH, 700 μL of 35% NH_3_ solution, and 1 mL of 20% AgNO_3_ in distilled water) for 30 min. The gel was washed twice for 15 min in distilled water and developed in 500 mL 1% citric acid containing 250 μL 37% formaldehyde.

### 2.5. Image Acquisition and Analysis

ImageQuant 300 (GE Healthcare) software was used for recording silver-stained gels images and analyzed with the PDQuest 8.0.1 software package (Bio-Rad, Hercules, CA, USA) to avoid false identification of proteins [15]. Spots were automatically detected on the basis of spot parameters such as the faintest, smallest, and largest spot on the gel scan. Images were filtered and edited to check possible errors. The intensity levels of the images are represented as the relative volume of the spots in each gel. Silver staining showed a dynamic range of 1 to 3 orders of magnitude according the software, and the weakest and strongest spots fell within this range. Only well-resolved spots were taken into account. 

### 2.6. Immunoblotting

Proteins were transferred to nitrocellulose membranes using the Trans-Blot^®^ Turbo^TM^ Transfer system and transfer pack (Bio-Rad). Transfer was carried out under the following conditions: 2.5 A-25 V for 20 min. Membranes were blocked in Tris-buffered saline (TBS) containing 5% powdered milk substitute. The membrane was incubated in primary antiserum (pooled sera from *Anisakis*-allergic patients diluted 1:20) overnight. Membranes were then washed in TBST and incubated in anti-human IgE monoclonal antibody diluted 1:1000 (E21A11, Ingenasa, Madrid, Spain) for 3 h after which the membranes were washed in TBST. The blots were then incubated in goat anti-mouse IgG-AP (1:30,000) for one hour followed by repeated washing in TBST. The antibody complex was detected by adding BCIP/NBT solution (Amresco, Solon OH, USA). The reaction was stopped by immersing the membranes in distilled water. Sera from *Anisakis*-nonallergic patients were used as a negative control. 

### 2.7. In-Gel Protein Digestion and Sample Preparation

The corresponding immuno reactive bands and spots in the replicated gels were excised manually from the silver-stained gel, deposited in eppendorfs, and processed automatically in a Proteineer DP (Bruker Daltonics, Bremen, Germany). The digestion protocol was based on [16] with minor changes: gel plugs were first washed with 50 mM ammonium bicarbonate and then with ACN prior to reduction with 10 mM DTT in 25 mM ammonium bicarbonate solution, and alkylation carried out with 55 mM IAA in 50 mM ammonium bicarbonate solution. Gel pieces were further rinsed with 50 mM ammonium bicarbonate and ACN and dried under a stream of nitrogen. 

A final concentration of 16 ng/μL of Proteomics Grade Trypsin (Sigma Aldrich, St. Louis, MO, USA) in 25% ACN/50 mM ammonium bicarbonate solution was added and the digestion took place at 37 °C for 6 hr. The reaction was stopped by adding 0.5% TFA for peptide extraction. The tryptic eluted peptides were dried by speed-vacuum centrifugation and resuspended in 4 µL of MALDI solution. A 0.8 μL aliquot of each peptide mixture was deposited onto a 386-well OptiTOF^TM^ Plate (SCIEX, Foster City, CA, USA) and allowed to dry at room temperature. A 0.8 µL aliquot of matrix solution (3 mg/mL α-Cyano-4-hydroxycinnamic acid in 30% ACN/15% isopropanol/0.1% TFA) was then deposited onto the dried digest and allowed to dry at room temperature. 

### 2.8. MALDI Peptide Mass Fingerprinting, MS/MS Analysis and Database Mining

For MALDI-TOF/TOF analysis, samples were automatically acquired in an ABi 4800 MALDI TOF/TOF mass spectrometer (SCIEX, Foster City, CA, USA) in positive ion reflector mode (voltage was 25 kV to MS acquisition and 1 kV to MSMS). The obtained spectra were stored into the ABi 4000 Series Explorer Spot Set Manager. PMF and MS/MS fragment ion spectra were smoothed and corrected to zero baseline using routines embedded in ABi 4000 Series Explorer Software v3.6. Each PMF spectrum was calibrated with the mass signals of trypsin autolysis ions to reach a typical mass measurement accuracy of <25 ppm. Known trypsin and keratin mass signals, as well as potential sodium and potassium adducts (+21 Da and +39 Da) were removed from the peak list. To submit the combined PMF and MS/MS data to MASCOT software v.2.6.1 (Matrix Science, London, UK), GPS Explorer v4.9 was used, searching in *Ansakis simplex* complex protein database from Uniprot [Uniprot 20200511 (25691sequences; 6802157 residues]. The following search parameters were used: enzyme, trypsin; allowed missed cleavages, 1; carbamidomethyl cysteine as fixed modification by the treatment with iodoacetamide; variable modifications, oxidation of methionine; mass tolerance for precursors was set to ± 25 ppm and for MS/MS fragment ions to ± 0.2 Da. The confidence interval for protein identification was set to ≥95% (*p* < 0.05) and only peptides with an individual ion score above the identity threshold were considered correctly identified. The mass spectrometry proteomics data have been deposited to the ProteomeXchange Consortium via the PRIDE [17] partner repository with the dataset identifier PXD019580 and 10.6019/PXD019580. 

## 3. Results 

### 3.1. Silver Staining and Western Blotting

**Figure 1:**
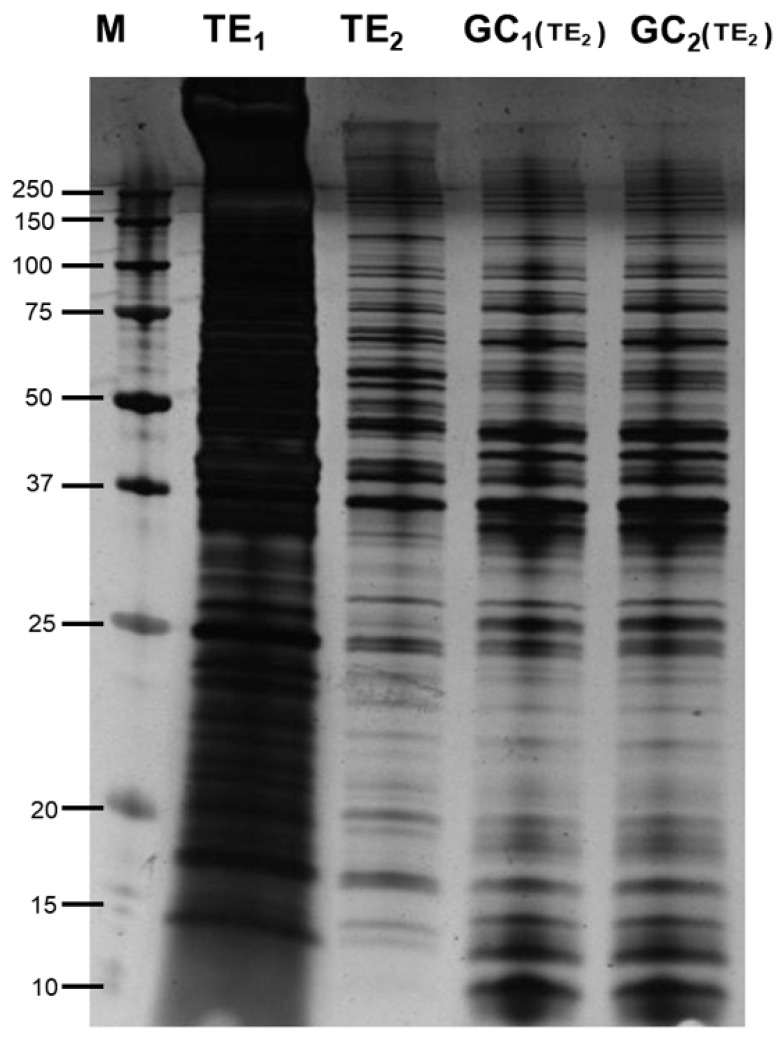
Monochromatic silver staining of total extracts and gland cell extracts of *A. simplex* (GC = gland cells), (TE = total extracts); (TE_1_ = denaturing), (TE_2_ = not denaturing). GC_1_ and GC_2_ are two technical replicates with phosphate.

**Figure 2:**
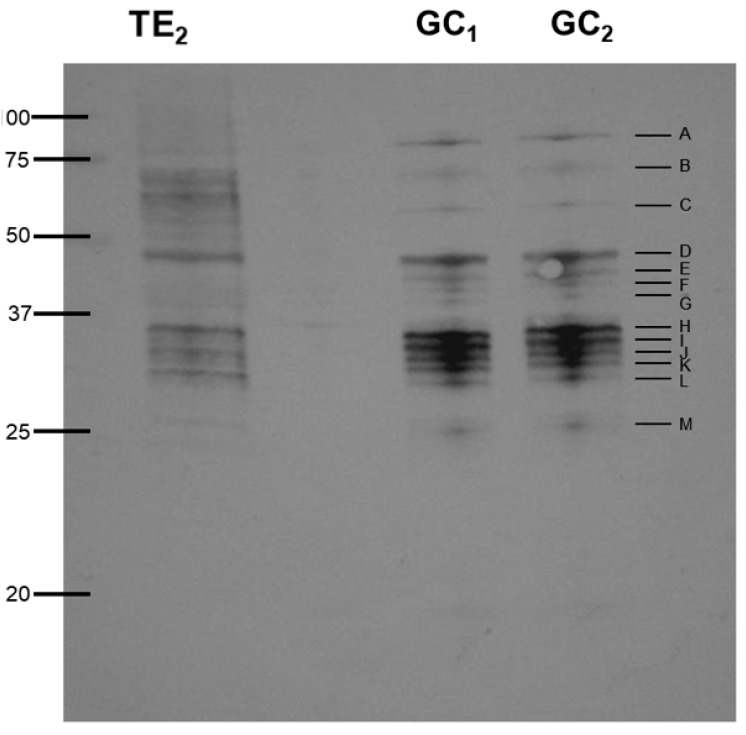
Western blot of total extracts and gland cell extracts probed with pooled serum of *Anisakis*-positive patients.

SDS-PAGE (1D) gels clearly demonstrate differences between the two protein extraction buffer systems used. The denaturing extraction buffer extracted more proteins (TE_1_) when compared to proteins extracted in phosphate buffer (TE_2_) probably due to the fact that the buffer is able to solubilize more proteins, however this method masks the bands and does not allow to see what are the western blot hybridized bands (Figure 1 and Figure 2). Differences are apparent between the total extracts depending of the applied method (T1 or T2) when same amount of protein is used; also, the same bands pattern is detected comparing total extracted proteins (TE_2_) and gland cells (GC_1_ an GC_2_) extracted in phosphate buffer when equal quantities of protein were loaded in each well. 

The small differences detected in Figure 1 suggest that other than the most abundant proteins were present in gland cell components as is detected in bands less 20 kD and higher 100 kD (this part was not immunoreactive). When blots were probed with pooled sera from *Anisakis*-allergic patients no differences are observed between the total extracts and the gland cells (Figure 2) although the binding of some proteins was very unclear observed in the total extract. Thirteen immunoreactive bands ranging from 25 to 80 kDa were observed in both gland cell extracts suggesting that the gland cells may be an important structure in the production of allergens. 

**Figure 3:**
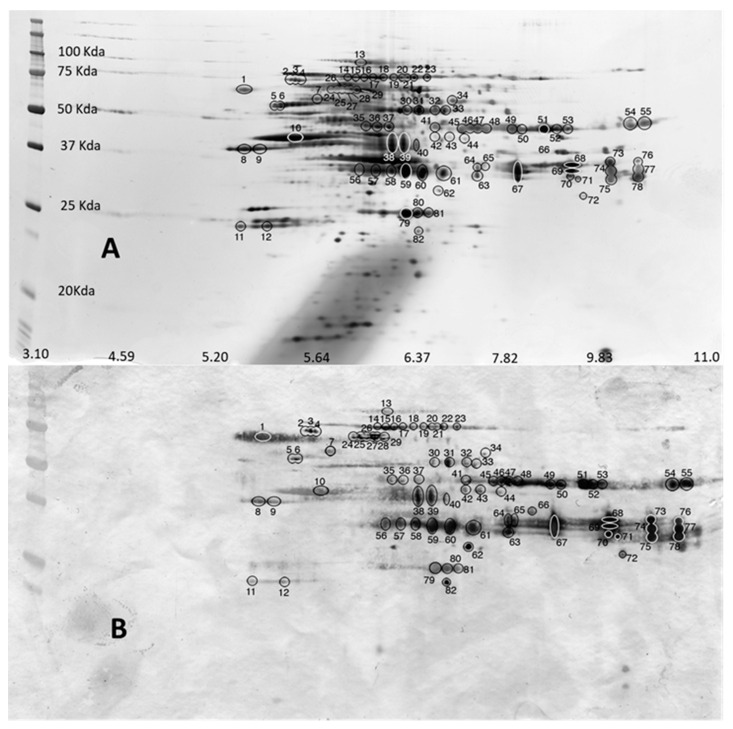
(**A**) 2-DE gel in which the corresponding proteins of western blots are highlighted. (**B**) Western blot showing the immunoreactive proteins

Selected 2-DE gels were incubated for western blotting and only unambiguously identified western blot spots in the 2-DE gels have been considered for proteomics identification (Figure 3A). Western blots spots were easily detected on the other replicated gels because of the high sensitivity range of silver staining (3 orders of magnitude) [18,19]. The 2-DE images of this master western blots displayed 82 spots (Figure 3B). Most of the spots were found between 100 kDa and 20 kDa and throughout the neutral to basic pI value range. The 82 blotting spots were apparently disposed in 14 different lines which seem coincident with the monodimensional western blots gels (Figure 2). 

### 3.2. MALDI Peptide Mass Fingerprinting, MS/MS Analysis and Database Mining

The obtained peptide sequences and homologies from each gel slice are shown in Table 1 (1-D and 2-D). For 2-DE experiment, only 27 spots have resulted in positive significant identification. All peptides gave significant matches to *Anisakis simplex* in the public databases (UniProt: https://www.uniprot.org) and other nematodes of the super family ascaradoidea, including *Ascaris suum* and *Toxocara canis*. In bands F, G, I, and M there are two proteins, while in band H there are three, being the expression of two and three loci respectively. The reason could be that the region where these bands are located is coincident with the not lineal gel concentration. The identification by 2-D electrophoresis allows resolving the mix of proteins. In this case, several proteoforms of the same protein are identified for locus eno (3 proteoforms), locus asim_10032 (5 proteoforms), locus asim_nas-13 (5 proteoforms), locus asim_14439 (6 proteoforms) and locus asim_14262 (2 proteoforms). There are also two proteoforms of the same protein from two different loci (locus asim_7120 and locus asim_19882). In total thirteen different proteins, as expression of sixteen different loci have been found to be immunoreactive to antibodies from allergenic patients (Table 2).

## 4. Discussion

The SDS-PAGE analysis of the proteins extracted from total nematodes and those extracted from the gland cells clearly show differences in protein banding. The total extract extracted with urea solubilizes a wider range of proteins; however, these proteins are completely denatured. Extracting the proteins in phosphate buffer would allow further study including enzyme activity assays (data not shown). A clear enrichment of proteins is observed in the gland cells (Figure 1). After western blotting, 13 immunoreactive bands were observed when the blots were probed with sera from *Anisakis* allergic patients. Only potential allergens were detected since anti-human IgE monoclonal was used in the experiment. Sera from *Anisakis* nonallergic patients showed no binding in western blot experiments. The nature of the esophagus and pharynx region, where many proteins have parasite function [20], grounds the hypothesis of this study. There is a mixture of proteins in the bands F to M and results were complemented by 2-D electrophoresis. In this case from 82 immunoreactive spots, only 27 produced significant positive identification results.

Excretory/secretory products (ES) from both animal and plant parasitic nematodes have been shown to be important in the parasitic process and can arrive from a number of structures including the amphids, cuticle, esophageal gland cells, ES system, and anus are therefore considered as potential origins of immunogenic proteins. 

Peptidase_M13_N domain-containing proteins (locus asim_19882 and locus asim_7120) are proteases found in many organisms, including mammals and bacteria. It is a soluble secreted endopeptidase with a broad range of physiological roles allowing a large substrate specificity [21].

A putative chitinase (locus asim_15940) was detected in bands E and G; its sequence characterizes it as glycoside hydrolase, family 19, catalytic [22]. Chitinases and chitinase-like proteins are thought to be important in immunomodulation during helminth infections. These molecules have also been shown to be secreted [23]. Chitinase found secreted by infective-stage juveniles of the sheep parasitic nematode, *Onchocerca volvulus* have been shown to be exclusively synthesized in the gland cells of the esophagus and stored as discrete secretory granules with a role in the early post-infective migration and/or development [23]. Other roles have been suggested for chitinases in animal parasitic nematodes including ensheathment in *Brugia malayi* once the parasite has entered the mosquito [24]. Chitinases have also been detected in the subventral esophageal glands of the plant parasitic nematode *Heterodera glicines* [25]. However, no clear function has yet been assigned. To date, most of the chitinases from nematodes would appear to belong to the class 18 family. Chitinases from mites are known to be highly glycosylated major allergens [26]. Chitinase proteins and inactive chitinase-like proteins are very important in mammalian allergy, asthma, and other Th2 type pathologies [27,28].

Lipase-like proteins (Lipase_3 domain-containing protein locus asim_15251) were identified in bands F and G. Lipases have been detected in the excretion/secretion products of nematodes including *Heligmosomoides polygyrus* [29]. Lipase-like proteins were detected in bands F and G, the best homologies coming from *T. canis*, *Wuchereria bancrofti*, and *Strongyloides ratti* and would appear to be more related to the class 3 lipases. Little information exists on the secretion of these molecules; however, sequence data is available due to a number of nematode genome projects in progress. Lipase-like proteins are known to be of allergenic importance with the majority being found in wasp and bee stings, and snake bites however only two class 3 lipases have been described as allergens “Rhi o” Lipase from *Rhizopus oryzae* [30] and “the l” Lipase from *Thermomyces lanuginosus* [31] from which sources of enzymes are to be incorporated into washing detergents (AllFam database of allergen families). 

Other proteases were identified between bands H-L and spots 57–73 with homology to Zinc Metalloproteases (astacin family) nas-13, and probably nas-15 (LOCUS nas-13 and LOCUS14439) due the phylogenetic relationships of astacins within nematodes [32]. These proteases have been shown to be present in the ES products of many animal and plant parasitic nematodes and these proteases are often developmentally regulated with differences between L3 and L4 feeding stage nematodes [33,34,35,36,37], therefore the presence of proteases in the gland cells of *A. simplex* is not surprising and confirms the results obtained by other authors [38] who identified secreted neutral proteases from *A. simplex* as circulating antigens. 

Protein lethal (2) essential for life (locus asim_12322) has been inferred by orthology as a *Drosophila melanogaster* protein [39]. It was identified in band M and spot 82; it can be considered as homologous of a small heat shock protein [40] with homology to OV25-1 from *Toxocara canis*. Small heat shock proteins have been cloned from a number of animal parasitic nematodes including *H. contortus* [41] *N. brasiliensis* [42], and *B. malayi* [43]. The role of these sHSP in parasitism is not very clear however in the trematode *Schistosome mansoni* sHSPs are immunogenic, producing a strong Th1-type immune response [44].

The structural protein Actin 3 was identified in band F. Other proteoforms of actins have been previously reported in *A. simplex* complex [9]. Proteins of the actin family have been detected in the ES products of different animal parasitic nematodes including *B. malayi* and *Dirofilaria immitis* [45,46] with a possible role in the acceleration of plasmin generation in the host [47].

One of the most interesting proteins identified is glyceraldehyde 3 phosphate dehydrogenase (locus GPDH) which belongs to the GADPH superfamily. According to the AllFam database of allergen families, GADPH is known to be a major allergen in the indoor mold *Aspergillus versicolor* [48] and a minor allergen (Tri a 34) for patients with baker’s asthma (IUIS Allergen Nomenclature Database). More recently GADPH has been described as a major allergen in rambutan-induced anaphylaxis [49] and it has been detected as a potential allergen in *A. pegreffii* [9].

SCP domain-containing protein (locus asim_10032 and locus asim_14262) are known *Ancylostoma* secreted proteins; they were identified in immunoreactive bands D and M. These proteins also known as venom allergen-like proteins (VALs) have become of interest in recent years. VALs belong to the SCP/TAPS family within the cysteine-rich secretory protein (CRISP) “superfamily” [50]. Many animal and plant parasitic nematodes are known to contain venom-like allergens [8]. Various roles have been proposed including the initiation and maintenance of the host-parasite relationships in the case of *Ancylostoma caninum* [51], invasion of the vertebrate host in the case of *B. malayi* [52], and establishment of plant-host parasite relationships in the plant parasitic nematode *Meloidogyne incognita*. In *Onchocerca volvulus*, the VAL protein Ov-ASP-1 was shown to be exclusively localized in the granules of the glandular esophagus in L3 stage larvae [53], therefore it is not surprising that we detect this protein in the gland cell extracts of *A. simplex*.

Uncharacterized protein from locus asim_540 could be considered as phosphoenolpyruvate carboxykinase due its molecular function and the biological process in which it participates, identified in band B is an enzyme belonging to the lyase protein family and is involved in the metabolic pathway of gluconeogenesis converting oxaloacetate into phosphoenolpyruvate and carbon dioxide. It is found in two forms, cytosolic and mitochondrial. In this study the molecular weight is predicted to be approximately 72 kDa, this is concurrent with the literature, with the homologous protein in *Haemonchus contortus* and *A. suum* which have molecular weights of 75–80 kDa and 70 kDa respectively [54,55]. To date, no information is available as to a possible role in parasitism or its presence in ES products of nematodes. 

Glycerol-3-phosphate dehydrogenase (locus14675) is a highly conserved protein found on the outer surface or as a secretory product of pathogenic organisms. It has a role as a virulence factor in a large number of pathogens from unicellular to small invertebrates [56]. Increased GPDH activity, especially GPD2, leads to an increase in glycerol production [57]. This is a mechanism of protection of nematodes against desiccation and freeze [58,59].

Uncharacterized protein from asim_locus16599 (domains DUF4139 and DUF4140) is a member of a conserved hypothetical protein CHP02231 whose function is unknown. DUF families within the Pfam database represent over 22% of known families [60].

CO esterase domain-containing protein (asim_locus12965) is a member of the Carboxylesterase family encompassing hydrolytic enzymes which are widely distributed along evolutionary branches with different catalytic function that share a common folding [61]. This family is a mixture of specialized enzymes with specific substrates, but also includes less selective members with broad and often overlapping ranges of substrates. It is thought to have a special role in insect resistance to pesticides [62]. Carboxylesterases are widely distributed in nature. Most of them participate in the metabolism of toxins or drugs producing carboxylates which are conjugated by other enzymes to increase solubility and excreted [63]. No more specific characteristics can be obtained from our results, but the carboxylesterase family includes a number of proteins with different substrate specificities, such as acetylcholinesterase. The role of acetylcholinesterase in nematode parasitism is well known and studied. Many parasitic nematodes of the alimentary tract release acetylcholinesterases which are thought to reduce the contractions of the alimentary tract and hence prevent parasite expulsion. Another role for secreted acetylcholinesterases may be immune modulation and reduction of inflammation in the site of the nematode infection [64]. Recently acetylcholinesterase was found in *A. simplex* L3 larva ES products [65,66]. 

Enolase (allergen Ani s Enolase) from asim_locus eno is found in the cytosol as part of phosphopyruvate hydratase complex (synonym of enolase complex). Enolases form a large superfamily with many proteoforms. The protein in this study was demonstrated to be *Anisakis simplex* allergen [67]. This and the other two enolase proteoforms were also found to be immunoreactive [9]. Although most enolases are found in the cytosol, cell surface-associated enolase is reported to promote pathological alterations and penetration of host tissues by pathogens and tumor cells [68]. In the case of nematode *Steinernema glaseri*, this protein was reported as a surface enolase, localized to both the nematode cuticle and the surface coat, which was secreted to the hemolymph of the host insect conferring host immune suppression [69].

Most of the proteins described have been shown to be present in the ES products of animal and plant parasitic nematodes indicating that the collected gland cells contain molecules which can actively be secreted into the host and therefore could be potential allergens accordingly to the immunoreactivity they have shown. Functional analysis using STRING (string-db.org) showed no relationships (88%, data not shown) except for Enolase and Glyceraldehyde-3-phosphate dehydrogenase which usually are coexpressed in *Caenorhabditis elegans*. We have detected 5 proteins (glyceraldehyde 3 phosphate dehydrogenase, chitinase, lipase_3 domain-containing protein, SCP domain-containing protein, and enolase) which are considered to be allergens ([70], allergome section in www.mncn.csic.es), and in this work for the first time described from *Aniskais simplex s.s*. which are expressed in their gland cells. Three proteins which were previously detected as immunoreactive in whole-body protein extracts [9] are also detected in gland cells (actin, glyceraldehyde-3-phosphate dehydrogenase, and enolase). The next stage will be to clone and overexpress candidate proteins including the eight other proteins in this study and determine their allergenic potential. 

## Figures and Tables

**Table 1:** Identified immunoreactive protein markers using MASCOT database searches. For 1-D experiments the results are for the two replicates (GC_1_, GC_2_) except for bands L and M which were negative for GC_2_.

	Protein	UniProt Accession	Taxonomy	Matched Peptides	(MS/MS)	Score	Gene
**1-D bands (Figure 1 and Figure 2)**							
A	Peptidase_M13_N domain-containing protein	A0A0M3KHN4	*Anisakis simplex*	10	R.ILTNYIVWR.Y R.YDDILQDFLR.V K.WAQNYWFR.K	133/175	ASIM_LOCUS19882
B	Uncharacterized protein	A0A0M3IZF7	*Anisakis simplex*	21/22	R.PFMGYNFGR.Y R.MGAAVLHELTR.T R.RPEGVPLVFESR.D R.LYAINPEAGFFGVAPGTSHK.T	205/238	ASIM_LOCUS540
C	unidentified			4	R.SFLGVPFAEPPIGENR.F	51/56	N/A
D	SCP domain-containing protein predicted	A0A0M3JRE8	*Anisakis simplex*	12/13	R.RAQEYAER.C R.NMYYLDYDCDLER.R R.STNLCIAPGYTAPEPNPR.P	193/255	ASIM_LOCUS10032
E	Uncharacterized protein	A0A0M3K6E2	*Anisakis simplex*	20	R.SKEEADDCFYR.G K.EFVALNEDNQKR.C K.TADGFYTDCYFGR.G K.CWPYSYEAFVIAAR.Y R.FGTSSPNSVYTPEENTR.R R.FGTSSPNSVYTPEENTRR.D R.DVAAFFAHAIQETGANDASVYTGR.S	469/530	ASIM_LOCUS15940
F	Lipase_3 domain-containing protein	A0A0M3K4F3	*Anisakis simplex*	10	K.HTELVPYSFR.V K.IIADHASYFDR.V K.NVLPSDEKWEVVER.I R.GTTTSSQLFLQGAGAISGR.A	120/134	ASIM_LOCUS15251
	Actin 3	A0A0B4SVN7	*Anisakis simplex*	9/10	K.QEYDESGPSIVHR.K K.SYELPDGQVITIGNER.F	113/119	N/A
G	Lipase_3 domain-containing protein	A0A0M3K4F3	*Anisakis simplex*	7/6	K.HTELVPYSFR.V	71/68	ASIM_LOCUS15251
G	Uncharacterized protein	A0A0M3K6E2	*Anisakis simplex*	11	K.TADGFYTDCYFGR.G	80	ASIM_LOCUS15940
H	Glyceraldehyde-3-phosphate dehydrogenase	A0A097IYH5	*Anisakis simplex*	4	R.VPTPDVSVVDLTCR.L K.LISWYDNEFGYSCR.V	96	GPDH
H	Hemoglobin	A0A1W7HP35	*Anisakis simplex*	10	---	58	N/A
H	Uncharacterized protein	A0A0M3JQQ1	*Anisakis simplex*	2	R.VLTDAIYLISHIDGTTR.M	147	ASIM_LOCUS9737 (very sort seq)
H	Metalloendopeptidase	A0A0M3K299	*Anisakis simplex*	9/7	R.VLFENINPPMR.C K.SSNYFLTDEDFER.A	142/94	ASIM_LOCUS14439
I	Metalloendopeptidase	A0A0M3K299	*Anisakis simplex*	10/9	R.VLFENINPPMR.C K.SSNYFLTDEDFER.A	180/156	ASIM_LOCUS14439
I	Metalloendopeptidase	A0A3G5BC99	*Anisakis simplex*	10/9	R.TGFSLNDVR.K	102/94	nas-13
J	Metalloendopeptidase	A0A0M3K299	*Anisakis simplex*	8	R.VLFENINPPMR.C K.SSNYFLTDEDFER.A	92/134	ASIM_LOCUS14439
K	Metalloendopeptidase	A0A0M3K299	*Anisakis simplex*	9	R.DPNALWPK.G R.VLFENINPPMR.C K.SSNYFLTDEDFER.A	139/126	ASIM_LOCUS14439
L	Metalloendopeptidase	A0A0M3K299	*Anisakis simplex*	10	R.DPNALWPK.G R.ADRDDYVR.V R.VLFENINPPMR.C	138	ASIM_LOCUS14439
M	Protein lethal(2) essential for life (inferred by orthology to a D. melanogaster protein)	A0A0M3JX08	*Anisakis simplex*	8	R.SIPIQAAPR.Q K.QNQQLPAAR.S R.YAIVPPTFER.A	96	ASIM_LOCUS12322
M	SCP domain-containing protein	A0A0M3K1U4	*Anisakis simplex*	5	K.QVNVVCEYR.N	71	ASIM_LOCUS14262
**2-D spots (Figure 3)**							
16	Peptidase_M13_N domain-containing protein	A0A0M3JI80	*Anisakis simplex*	7	K.YDLTNLLIHTSLTR.A	78	ASIM_LOCUS7120
16	Peptidase_M13_N domain-containing protein	A0A0M3KHN4	*Anisakis simplex*	4	R.ILTNYIVWR.Y	70	ASIM_LOCUS19882
29	Uncharacterized protein	A0A0M3K8A1	*Anisakis simplex*	21	K.SASIVNEQR.I R.FVCSLGVDPGIK.L K.LAAEIGALQHDER.Y	214	ASIM_LOCUS16599
31	CO esterase domain-containing protein	A0A0M3JYK8	*Anisakis simplex*	6	R.ADVFLGVPYAQPPVGALR.F	101	ASIM_LOCUS12965
35	Enolase	Q8MU59	*Anisakis simplex*	8	R.AAVPSGASTGVHEALELR.D K.STLNIQLVGDDLTVTNR.E	101	eno
36	Enolase	Q8MU59	*Anisakis cimplex*	16	R.AAVPSGASTGVHEALELR.D K.STLNIQLVGDDLTVTNR.E R.YGLDATAVGDEGGFAPNIQDNR.E	276	eno
37	Enolase	Q8MU59	*Anisakis simplex*	7	R.AAVPSGASTGVHEALELR.D	66	eno
46	SCP domain-containing protein	A0A0M3JRE8	*Anisakis simplex*	6	R.RAQEYAER.C R.STNLCIAPGYTAPEPNPR.P	59	ASIM_LOCUS10032
50	SCP domain-containing protein	A0A0M3JRE8	*Anisakis simplex*	7	R.RAQEYAER.C R.STNLCIAPGYTAPEPNPR.P	84	ASIM_LOCUS10032
51	SCP domain-containing protein	A0A0M3JRE8	*Anisakis simplex*	10	R.RAQEYAER.C R.NMYYLDYDCDLER.R R.STNLCIAPGYTAPEPNPR.P	159	ASIM_LOCUS10032
52	SCP domain-containing protein	A0A0M3JRE8	*Anisakis simplex*	9	R.RAQEYAER.C R.NMYYLDYDCDLER.R R.STNLCIAPGYTAPEPNPR.P	148	ASIM_LOCUS10032
54	SCP domain-containing protein	A0A0M3JRE8	*Anisakis simplex*	9	R.RAQEYAER.C R.NMYYLDYDCDLER.R R.STNLCIAPGYTAPEPNPR.P	136	ASIM_LOCUS10032
57	Metalloendopeptidase	A0A3G5BC99	*Anisakis simplex*	9	R.TGFSLNDVR.K	97	nas-13
58	Metalloendopeptidase	A0A3G5BC99	*Anisakis simplex*	6	R.TGFSLNDVR.K	75	nas-13
59	Metalloendopeptidase	A0A3G5BC99	*Anisakis simplex*	15	R.TGFSLNDVR.K R.TGFSLNDVRK.I K.GVVIHELMHALGIQHEQSR.T	190	nas-13
60	Metalloendopeptidase	A0A3G5BC99	*Anisakis simplex*	10	R.TGFSLNDVR.K R.TGFSLNDVRK.I K.NGKPTIVALEPNR.N	103	nas-13
61	Metalloendopeptidase	A0A3G5BC99	*Anisakis simplex*	7	R.TGFSLNDVR.K	70	nas-13
63	Glycerol-3-phosphate dehydrogenase	A0A0M3K2U5	*Anisakis simplex*	10	MVSLRNAIVGFTR.A	47	ASIM_LOCUS14675
64	Metalloendopeptidase	A0A0M3K299	*Anisakis simplex*	8	R.ADRDDYVR.V R.VLFENINPPMR.C R.VLFENINPPMR.C K.SSNYFLTDEDFER.A	96	ASIM_LOCUS14439
67	Metalloendopeptidase	A0A0M3K299	*Anisakis simplex*	8	R.VLFENINPPMR.C R.VLFENINPPMR.C K.SSNYFLTDEDFER.A	139	ASIM_LOCUS14439
68	Metalloendopeptidase	A0A0M3K299	*Anisakis simplex*	10	R.VLFENINPPMR.C R.VLFENINPPMR.C K.SSNYFLTDEDFER.A K.SSNYFLTDEDFERAR.S	169	ASIM_LOCUS14439
69	Metalloendopeptidase	A0A0M3K299	*Anisakis simplex*	7	R.ADRDDYVR.V K.SSNYFLTDEDFER.A	82	ASIM_LOCUS14439
70	Metalloendopeptidase	A0A0M3K299	*Anisakis simplex*	8	R.ADRDDYVR.V R.VLFENINPPMR.C R.VLFENINPPMR.C	90	ASIM_LOCUS14439
73	Metalloendopeptidase	A0A0M3K299	*Anisakis simplex*	4	R.ADRDDYVR.V K.SSNYFLTDEDFER.A	84	ASIM_LOCUS14439
79	SCP domain-containing protein	A0A0M3K1U4	*Anisakis simplex*	9	K.QVNVVCEYR.N K.YGVTASEAWWSELKR.V R.HAGENIFASSTTGSLGDLGK.Y	247	ASIM_LOCUS14262
80	SCP domain-containing protein	A0A0M3K1U4	*Anisakis simplex*	6	K.QVNVVCEYR.N	75	ASIM_LOCUS14262
81	SCP domain-containing protein	A0A0M3K1U4	*Anisakis simplex*	4	K.QVNVVCEYR.N	53	ASIM_LOCUS14262
82	Protein lethal(2)essential for life	A0A0M3JX08	*Anisakis simplex*	9	R.SIPIQAAPR.Q R.YAIVPPTFER.A	88	ASIM_LOCUS12322

**Table 2:** Identified and assigned proteins based on higher score of Table 1, with important molecular functions and biological processes. (*) are allergen proteins.

Gene	Protein	UniProt Accession	1-D band	2-D spot	Molecular function	Biological Process
ASIM_LOCUS19882	Peptidase_M13_N domain-containing protein	A0A0M3KHN4	band A	16	Catalysis of the hydrolysis of internal, alpha-peptide bonds in a polypeptide chain	Metalloendopeptidase activity
ASIM_LOCUS7120	Peptidase_M13_N domain-containing protein	A0A0M3JI80		16		
ASIM_LOCUS540	Uncharacterized protein	A0A0M3IZF7	band B		phosphoenolpyruvate carboxykinase (GTP) activity	gluconeogenesis
ASIM_LOCUS10032	SCP domain-containing protein (*)	A0A0M3JRE8	band D	46, 50, 51, 52, 54	Unclear function. Venom allergens of some insects and Scolopendra	Belongs to the CRISP family (cysteine-rich secretory proteins)
ASIM_LOCUS14262	SCP domain-containing protein (*)	A0A0M3K1U4	band M	79, 80, 81		
ASIM_LOCUS15940	Uncharacterized protein (*)	A0A0M3K6E2	bands E, G		chitinase activity	cell wall macromolecule catabolic process
ASIM_LOCUS15251	Lipase_3 domain-containing protein (*)	A0A0M3K4F3	bands F, G		hydrolase activity	lipid metabolic process
N/A	Actin 3	A0A0B4SVN7	band F		ATP binding	Cytoskeleton
ASIM_LOCUS14439	Metalloendopeptidase	A0A0M3K299	bands H, I, J, K, L	64, 67,68, 69, 70, 73	Metalloprotease	Metalloendopeptidase activity
nas-13	Metalloendopeptidase	A0A3G5BC99	band I	57, 58, 59, 60, 61		
GPDH	Glyceraldehyde-3-phosphate dehydrogenase (*)	A0A097IYH5	band H		Catalytic activity (Oxidoreductase)	glucose metabolic process (Glycolisis)
ASIM_LOCUS14675	Glycerol-3-phosphate dehydrogenase	A0A0M3K2U5		63	Catalytic activity (calcium ion binding)	Oxydation-reduction process
ASIM_LOCUS12322	Protein lethal(2)essential for life	A0A0M3JX08	band M	82	prevent the precipitation of denatured proteins and to increase cellular tolerance to stress	Alpha crystallin/Small heat shock protein, animal type
ASIM_LOCUS16599	Uncharacterized protein	A0A0M3K8A1		29	Unknown function	From a family of proteins over 500 amino acids in *Caenorhabditis elegans* and several bacteria
ASIM_LOCUS12965	CO esterase domain-containing protein	A0A0M3JYK8		31	Unknown function	
eno	Enolase (allergen Ani s Enolase) (*)	Q8MU59		35, 36, 37	magnesium ion binding Phosphopyruvate hydratase activity	Glycolytic process
